# Mutual regulation of lactate dehydrogenase and redox robustness

**DOI:** 10.3389/fphys.2022.1038421

**Published:** 2022-11-04

**Authors:** Yijun Lin, Yan Wang, Pei-feng Li

**Affiliations:** Xiamen Cardiovascular Hospital, Xiamen University, Xiamen, China

**Keywords:** lactate, lactate dehydrogenases, redox, ROS, post-translational modification

## Abstract

The nature of redox is electron transfer; in this way, energy metabolism brings redox stress. Lactate production is associated with NAD regeneration, which is now recognized to play a role in maintaining redox homeostasis. The cellular lactate/pyruvate ratio could be described as a proxy for the cytosolic NADH/NAD ratio, meaning lactate metabolism is the key to redox regulation. Here, we review the role of lactate dehydrogenases in cellular redox regulation, which play the role of the direct regulator of lactate–pyruvate transforming. Lactate dehydrogenases (LDHs) are found in almost all animal tissues; while LDHA catalyzed pyruvate to lactate, LDHB catalyzed the reverse reaction . LDH enzyme activity affects cell oxidative stress with NAD/NADH regulation, especially LDHA recently is also thought as an ROS sensor. We focus on the mutual regulation of LDHA and redox robustness. ROS accumulation regulates the transcription of LDHA. Conversely, diverse post-translational modifications of LDHA, such as phosphorylation and ubiquitination, play important roles in enzyme activity on ROS elimination, emphasizing the potential role of the ROS sensor and regulator of LDHA.

## Introduction

The nature of redox is electron transfer. Because electron transfer between matters is associated with energy release or absorption, redox process, to some extent, is the process of energy metabolism. The electron transport among proteins brings reductive power, together with energy transfer. A metabolic electron-transfer (ET) system catalyzes a complex network of biotransformations of organic compounds, while it is mainly NAD(P)H-dependent (Iyanagi, 2019). The NAD(P)H-dependent metabolic ET systems include three steps, where two electrons are transferred to the final metal-contained center. In this way, excess accumulation of NAD(P)H leads to reductive stress, which may be utilized by NADPH oxidases to produce reactive oxygen species (ROS) for redox homeostasis maintenance (Handy and Loscalzo, 2012). As the coenzyme for oxidoreductases and the substrate of some enzymes, the NAD+/NADH redox couple is commonly known as a regulator of cellular energy metabolism, that is, of glycolysis and mitochondrial oxidative phosphorylation (Xiao et al., 2018).

Glycolysis and tricarboxylic acid cycle (TCA cycle) are common metabolic pathways in aerobic organisms. A molecule of glucose would be catabolized into two molecules of pyruvate through glycolysis, associated with the production of one NADH, while six NADHs are produced in the TCA cycle. With these essential metabolic pathways, redox homeostasis is also built up. For example, blockage of glycolysis by 2-DG increases intracellular ROS and results in autophagic cell death (Rashmi et al., 2018), while pyruvate kinase muscle 2 (PKM2) activation inhibits normal NK cell oxidative metabolism fueling (Walls et al., 2020).

Lactate is the “by-product” of glycolysis, which is transformed from pyruvate and gets away from TCA cycles (Brooks, 2020). Though once recognized as a metabolic waste, lactate is now viewed as an important fuel in energy homeostasis. Lactate production is catalyzed by lactate dehydrogenase A (LDHA), while LDHB catalyzes the reverse process. Due to its easy transportation inside and outside cells through monocarboxylate transporters (MCTs), lactate is abundant in circulation (Brooks, 2018). A recent study found that the circulatory turnover flux of lactate is the highest of all metabolites and a primary source of carbon for the TCA cycle and thus of energy (Hui et al., 2017), verifying that lactate can be the key regulator of metabolism. The pool of intracellular and circulatory lactate, mediated by LDH and MCT, participates in the maintenance of redox robustness.

## Lactate homeostasis: Part and parcel of redox robustness

Lactate is now recognized to play roles in maintaining energy and redox homeostasis. The regulation of redox robustness is mediated by intercellular and interorgan flows of lactate, while lactate production and consumption is a key to that (Brooks, 2020; Lagarde et al., 2021).

**FIGURE 1:**
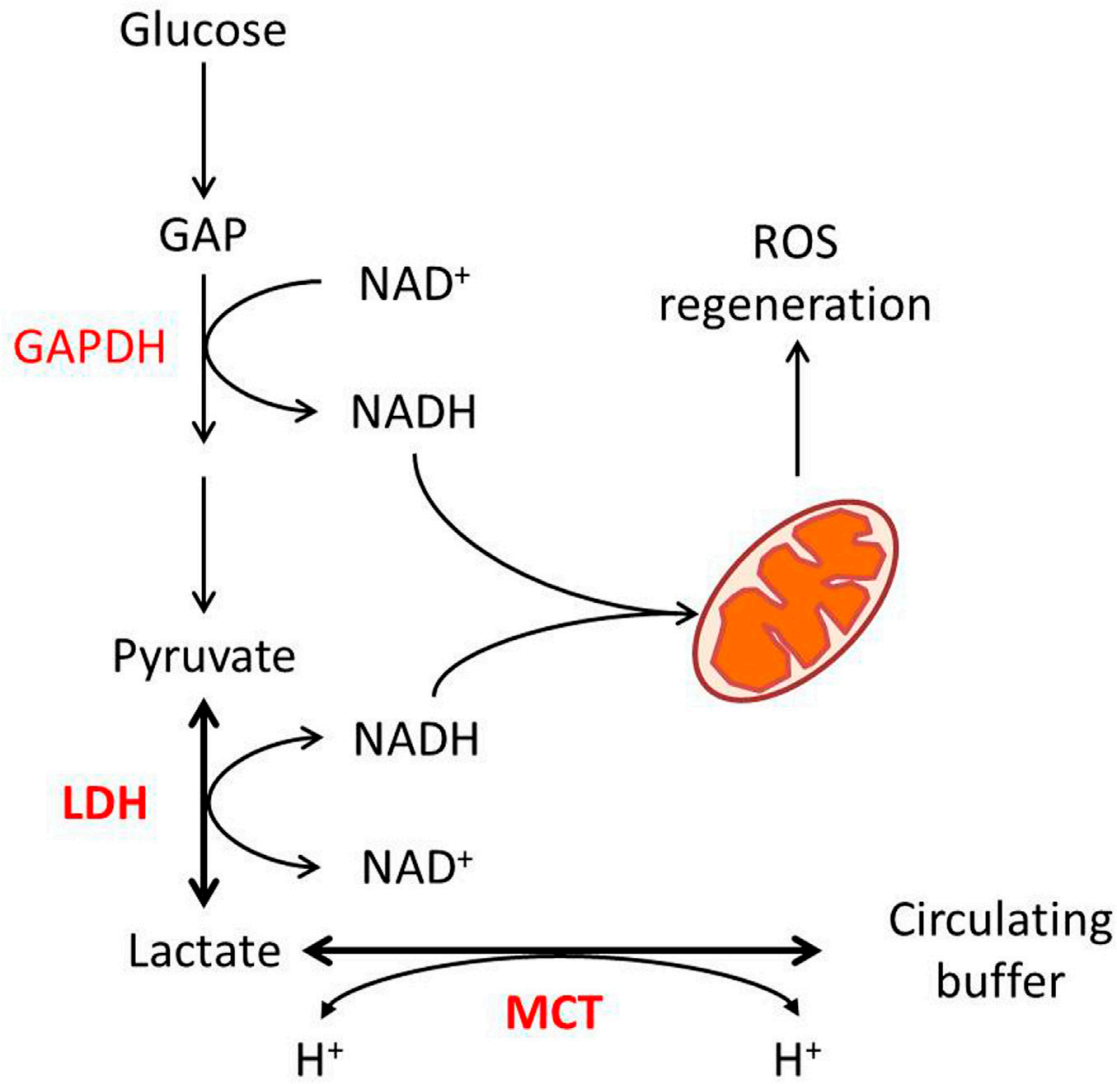
Lactate metabolism and redox homeostasis. Glycolytic flux from glucose to pyruvate generates NADH from NAD at the GAPDH reaction, while LDHA-catalyzed pyruvate to lactate consumes NADH. The pyruvate–lactate ratio in some conditions reflects the NAD–NADH ratio. MCT-mediated proton-dependent lactate transport maintains the balance between intracellular and circulated lactate.

In a glycolytic cell, lactate production and its export are critical for intracellular redox homeostasis. Two steps of glycolysis, the cellular G3P-to-1,3-BPG and lactate-to-pyruvate, both require NAD as a substrate, catalyzed by G3P dehydrogenase (GAPDH) and LDH, respectively (Figure 1). The reduction of pyruvate into lactate by LDH activity is associated with the oxidation of NADH, H^+^ into NAD (Lagarde et al., 2021). As a reversible reaction, the transformation between lactate and pyruvate, catalyzed by LDH, effectively buffers NAD and NADH in the cytoplasm. Some points show that while the LDH reaction approaches equilibrium, the cellular lactate/pyruvate ratio could be described as a proxy for the cytosolic NADH/NAD^+^ ratio (Mintun et al., 2004; Rabinowitz and Enerback, 2020).

In consideration of GAPDH’s lower Michaelis constant for NAD than LDH’s (Talaiezadeh et al., 2015), the export of lactate is required to enable the reaction to further move toward lactate production. Lactate transport is mainly mediated by four isoforms of MCT, which are also pyruvate and ketone body transporters (Halestrap and Wilson, 2012). Lactate transport mediated by MCT is associated with proton flux, depending on the electrochemical gradient (Halestrap, 2013). As for that, the transport of lactate through MCTs itself is also a way for maintenance of redox homeostasis. On the other hand, lactate consumption by oxidative cells is associated with proton uptake. In this case, the MCT–LDH reaction sequence would align the cytosolic NAD/NADH ratio to the circulating lactate, maintaining systemic redox robustness (Figure 1).

Lactate shuttle theory describes the roles of lactate in the delivery of oxidative and gluconeogenic substrates (Brooks, 2018). This theory not only elucidates lactate transport between cells but also between organelles. In oxidative cells, the TCA cycle and oxidative phosphorylation (OXPHOS) happening in mitochondria supply most of the energy for cell proliferation, which demands effective uptake and metabolism of lactate. Mitochondrial activity in some conditions could correspond to an electron dissemination process, and the electron load reflects the oxidative capacity of mitochondria. Some investigators put forward the “mitochondrial lactate oxidation complex” (mLOC) to describe several essential components of lactate oxidation in mitochondria, including MCT, its membrane chaperone basigin, LDH, and cytochrome oxidase (Brooks, 2018). This system has been reported in different tissues, such as the liver and muscle (Hashimoto et al., 2006; Passarella et al., 2014). However, it is controversial whether MCT is abundant in the inner membrane of mitochondria (Corbet et al., 2018; Rabinowitz and Enerback, 2020). Anyway, the lactate shuttle into mitochondria brings electrons and substrate for the TCA cycle, affecting the balance of the NAD/NADH ratio in the intermembrane space of mitochondria. Some overwhelming changes in cell metabolic states, like the Warburg effect in cancer cells and the uncoupling of the state of glycolysis and mitochondrial metabolism, result in the disruption of mitochondrial oxidative balance and the over-production of ROS (Lu et al., 2015). Through this metabolic and redox regulation, lactate facilitates electron management processes by activating specific signaling pathways, such as triggering mitochondrial biogenesis in muscle cells and adipocytes (Hashimoto et al., 2007; Li G. et al., 2017), and even uncoupling protein 1 (UCP1) expression in adipocytes (Carriere et al., 2014). In addition, the redox state drives lactate flux to regulate mitochondrial Mg^2+^ dynamics (Daw et al., 2020). These effects, by mechanism, are the adaptive ways to alleviate redox pressure. Very interestingly, even though the aforementioned studies discussed showed that lactate regulates downstream pathways through NAD/NADH and ROS production, recent research showed lactate excretion is also strongly affected by mitochondrial NADH shuttle activity (Wang et al., 2022). The mutual relationship of lactate and redox state might be the core of cell metabolism.

## Lactate, redox, and cell survival

Lactate metabolism has been proven to be associated with cell proliferation and survival, which is partly mediated by redox homeostasis. High lactate production is found in cancer cells correlating with tumor recurrence and metastatic potential, known as aerobic glycolysis or the Warburg effect (Zhang et al., 2019). This efficient glycolysis in cancer cells has been proven to maintain cell proliferation. Different alterations in glycolytic function have been approved to intervene in the cancer process (Ganapathy-Kanniappan and Geschwind, 2013). Some point out that the Warburg effect causes alterations in mitochondrial redox potential, ultimately changing ROS generation to affect cell survival (Locasale and Cantley, 2011; Liberti and Locasale, 2016). More importantly, anticancer therapies, such as ionizing radiation and several chemotherapeutic drugs, induce oxidative stress in targeted cells (Hirschhaeuser et al., 2011). Therefore, lactate accumulation could induce resistance to radiation and may cause chemoresistance (Sattler and Mueller-Klieser, 2009). Lactate pre-treatment reduces cancer cell death induced by oxidative stress and delays aging-evoked phenotypes in *C. elegans* (Tauffenberger et al., 2019). Different inhibitors of LDH, such as FX-11 and dichloroacetate (DCA), significantly increase oxidative stress, resulting in tumor cell death (Le et al., 2010; Haugrud et al., 2014). Targeting the lactate transporter MCT1 also increases the cell oxidative stress to the ferroptosis of HCC cells (Zhao et al., 2020). The Warburg effect also presents in some anabolism-activated tissues. In T cells, extracellular lactate correlates well with cell proliferation (Grist et al., 2018), which suggests immune-suppressive signaling of Treg cells in the tumor microenvironment (Watson et al., 2021). Further reports suggest that lactate leads to regulation of post-GAPDH glycolytic intermediates and T cell proliferation *via* the NAD(H) redox state (Quinn et al., 2020). In efficient glycolytic organs like adipose tissues, alteration of lactate transport promotes apoptosis of adipocytes, resulting in severe insulin resistance (Lin et al., 2022). All the research studies show that the redox homeostasis maintained by lactate decides cell fate.

## LDH: Structure and location

The lactate/pyruvate ratio, the representation of cellular redox robustness, is directly regulated by LDHs. LDH is a family of 2-hydroxyacid oxidoreductases that is found in almost all animal tissues and microorganisms, whose roles in metabolism have been well-established since 1932 (Granchi et al., 2010). Human LDH is a family of tetrameric isozymes (Neilands, 1952), and there are at least six isozymes of LDH. It is now recognized that active LDH is a homo- or hetero-tetramer assembled from two types of subunit: LDHA (M) and LDHB (H), encoded by separate genes located on chromosomes 11p15.4 and 12p12.2-p12.1 (Krieg et al., 1967; Augoff et al., 2015). The combinations of LDHA and LDHB result in five major isoforms of LDH (LDH1-5), with an increased composition ratio of LDHA. A third subunit, LDHC, likely a duplicate of LDHA, forms a testis-specific isoform known as LDH-6 (Goldberg, 1971).

LDHA and LDHB enzymes possess about 75% amino acid sequence identity, signifying significant similarity of overall structures except the residues forming substrate-binding pockets (Bellamacina, 1996). Despite the negligible structural diversity, dissimilarity in the kinetic properties of each form is pronounced. The overall turnover rate is two-fold higher for LDHA than for LDHB, whereas the LDHB shows about a three-fold increase in the ability to bind pyruvate (Granchi et al., 2010). These differences determine that LDHA mainly converts pyruvate to lactate and transforms NADH to NAD^+^ (Feng et al., 2018). On the contrary, LDHB protein kinetically favors the lactate-to-pyruvate conversion. Due to the differences in function, the distribution pattern of LDHs depends on the metabolic state. For example, LDH-5 and LDH-4 are found especially in anerobic tissues, like the liver, while LDH-1 strongly predominates in the cardiac muscle with principal aerobic metabolism (Cameron et al., 2004; Granchi et al., 2010).

**FIGURE 2:**
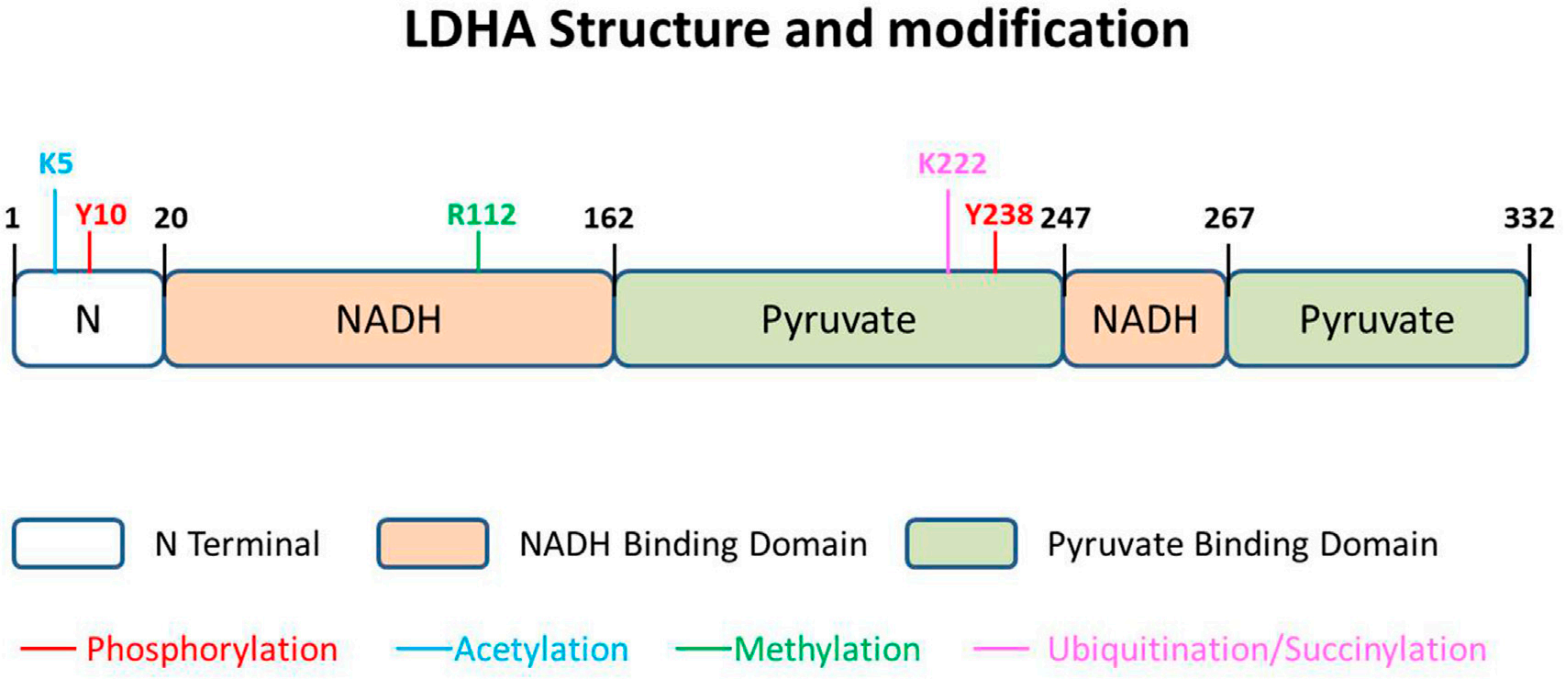
LDHA structure and modification. The 332-amino acid protein could be described as having three parts, N terminal domain, NADH-binding domain, and pyruvate-binding domain. There are five kinds of modifications that have been reported in different sites of LDHA, namely, phosphorylation, acetylation, methylation, ubiquitination, and succinylation.

LDHA contains 332 amino acids, usually existing as a tetramer (LDH-5) (Figure 2). As the most effective isoform in the family to catalyze pyruvate to lactate, most studies on this family focus on LDHA, and aberrant activation of LDHA has been found to be closely related to diverse cancers (Fantin et al., 2006; Le et al., 2010). LDHA is mainly located in the cytoplasm, acting as the glycolysis regulator through the pyruvate/lactate ratio, but it has also been reported inside mitochondria as part of mLOC (Brooks et al., 1999). More interestingly, about 0.5% of the total LDH-5 has been reported to be present in the nucleus, where it works as a single-stranded DNA-binding protein, participating in DNA duplication and transcription (Grosse et al., 1986). The location of LDHA has been investigated to be dependent on post-translational regulation in a stimulatory fashion (Cooper et al., 1984; Corbet et al., 2018).

## LDH and ROS

Due to the toxicity and metabolic affinity, the level of lactate in circulation is much higher than that of pyruvate, which shows the importance of LDH (Brooks, 2018). LDHA mediates lactate production and LDHB for consumption, becoming the key role in the lactate shuttle (Brooks et al., 1999). As circulated lactate is considered the main energy carrier in mammals (Hui et al., 2017), LDH, in a certain sense, mediates energy flux and balance of the whole body. Some reports have shown that LDH deficiency affects muscle stem cells and the tumor microenvironment through metabolic homeostasis (Theret et al., 2017; Watson et al., 2021), indicating the essential function of LDH in regulating energy flux.

Because of the enzyme activity, LDH is the direct handler of the lactate/pyruvate ratio, suggesting its correlation with redox homeostasis. LDHA induces the conversion of pyruvate to lactate, associated with NADH to NAD. This process supplies reducing power against reactive oxygen species (Brand and Hermfisse, 1997; Wu et al., 2021). More importantly, in glycolytic cells, the activity of LDHA ensures a low demand of mitochondria to produce ATP through oxidative phosphorylation, which avoids the elevation of mitochondrial ROS (Fantin et al., 2006; Jeong et al., 2006). A series of research studies have suggested high activity of LDHA to be a hallmark of cancer, where the regulation of ROS plays an important role (Le et al., 2010; Sheng et al., 2012; Feng et al., 2018). Therefore, LDHA expression or activity has become a valued target of cancer therapy. Diverse inhibitors of LDHA, such as FX-11, AZ-33, and NHI-2 (Granchi et al., 2013; Xie et al., 2014; Augoff et al., 2015), alter cancer cell survival through ROS accumulation, and knockdown of LDHA approaches similar effects (Le et al., 2010; Sheng et al., 2012).

As an enzyme of ROS regulation, LDHA recently is also thought of as an ROS sensor. Even though the expression of LDHA is not directly affected by ROS accumulation (Li S. et al., 2020), B7-H3, an immunoregulatory protein, could increase LDHA levels through HIF1a (Lim et al., 2016). Interestingly, ROS accumulation disrupts LDH-5 tetramer formation and promotes nuclear translocation of LDHA dimer (Liu et al., 2018). Nuclear LDHA produced α-hydroxybutyrate with activation of NRF2 downstream genes to protect cells from oxidative stress. This noncanonical enzyme activity highlights that LDHA could be recognized as a sensor of ROS accumulation, and its location may be the marker.

Due to the lower expression in most glycolytic tumor cells and metabolic organs like the liver and muscle than LDHA, LDHB escaped the attention of researchers for a long period. However, some recent studies show that LDHB also plays an essential role in redox regulation, particularly in oxidative cells. LDHB knockdown significantly decreases mitochondrial function and upregulates ROS production in UB/OC1 cells, which might be associated with age-related hearing loss (Tian et al., 2020). LDHB regulates mitochondrial respiration in leukemia but not in normal hematopoietic cells, also indicating the regulatory potential of redox state (Qing et al., 2021). Tumor-derived miR-375 downregulates macrophage LDHB for microenvironment remodeling, leading to a higher level of glycolysis and proliferation of cancer cells (Frank et al., 2021).

### ROS controls transcriptional regulation of LDHA

The promoter region of LDHA contains multiple elements for diverse transcription factor binding, such as HIF-1, MYC, KLF4, and FOXM1 (Feng et al., 2018). Thus, transcriptional regulation is usually thought to be the main regulatory mode of LDHA expression.

Hypoxia-induced factor 1(HIF-1) is a heterodimer binding to hypoxia-responsive elements (HRE) to activate targeted genes, which is one of the most important transcriptional factors of LDHA. Not only a responder to hypoxia, HIF is also a prominent determinant of the metabolic shift from glucose oxidation to aerobic glycolysis in cancer (Talaiezadeh et al., 2015). In hypoxic or stimulatory states, HIF-1α stabilization is augmented by mitochondrial ROS in an oxidant-dependent manner (Majmundar et al., 2010; Willson et al., 2022). HIF-1α guides the shift of glucose metabolism by promoting the expression of glycolytic enzymes and LDHA, which replenishes NAD^+^ for further metabolism in cancer cells (Gordan et al., 2007; Dong et al., 2022), resulting in the state of aerobic glycolysis. Through HIF-1a, LDHA is activated by ROS to maintain redox robustness.

MYC, a well-established oncogene, binds to the E-box in the promoter of LDHA, thus activating LDHA expression (Shim et al., 1997). MYC cooperates with HIF to activate PDK1 and LDHA and regulate mitochondrial biogenesis (Dang, 2012), but there is a lack of evidence that MYC would be directly regulated by ROS signaling.

FOXM1 belongs to the Forkhead transcriptional superfamily, found positively associated with LDHA in pancreatic cancer (Cui et al., 2014). Further investigation demonstrates that FOXM1 bound to the promoter of LDHA and promotes its transcription in pancreatic and gastric cancer to influence the glycolytic state of cancer (Cui et al., 2014; Jiang et al., 2015). It is noteworthy that FOXM1 is a typical transcriptional factor that responds to ROS. It has been reported that Ras-induced FOXM1 expression requires ROS (Park et al., 2009). Recently, studies show that NOX4 stimulates FOXM1 expression and further LDHA expression by increasing mitochondrial ROS in glioma (Su et al., 2021), and FABP4-associated ROS production could induce FOXM1 expression (Wu et al., 2018). Deletion of FOXM1 induces ROS accumulation and cell apoptosis (Kopanja et al., 2015), partly explained by the regulation of glycolytic gene expression.

KLF4 transferred into the nucleus exerts transcriptional regulation by binding to the GC box, 5′-CACCC-3′ sequence, of the promoter region (Feng et al., 2018). KLF4 was found negatively related to LDHA level by binding to −371 to −367 bp or −1,310 to −1,306 bp promoter region of LDHA (Shi et al., 2014). Some reports suggest that ROS may be responsible for eliciting a KLF4-mediated response to glucose starvation (Blum et al., 2021). Vascular smooth muscle cell migration and proliferation are also thought to be regulated by ROS-mediated KLF4 pathways (Huynh et al., 2022). Through KLF4, ROS regulates an indirect effect on LDHA expression. However, KLF4 activation-mediated ROS elimination could not be explained with LDHA function.

### Posttranslational modification of LDH

LDHA could also be modulated by posttranslational modification in specific amino acids. The posttranslational modification of LDHA, impacting protein stability and function, seems tighter and more direct in relationship to redox balance. Diverse modifications of LDHA have been reported, including phosphorylation, acetylation, and succinylation (Figure 2). In addition, a few studies report phosphorylation and acetylation of LDHB, performing a function in glycolytic regulation.

#### Phosphorylation predominantly promotes lactate production

Phosphorylation is the main and earliest discovered pattern of LDHA posttranslational modification, and most of the studies report its positive regulation on lactate-producing activity. It has been reported that the subcellular localization of LDHA appears to be dependent on the phosphorylation state of Y238 in 1984 (Cooper et al., 1984). Y10 is the most reported phosphorylated site of LDHA, which is shown upregulated in thyroid cancer tissues as compared to goiter (Kachel et al., 2015). The authors demonstrate that phosphorylation of LDHA occurs in an FGFR1-specific manner, which is an additional regulatory mechanism underlying the Warburg effect and lactate production in thyroid cancer cells. Further explorations show that an adenylate kinase, hCINAP, promotes FGFR1-catalyzed LDHA Y10 phosphorylation in CRC (Ji et al., 2017) and an lncRNA, HULC, modulates the phosphorylation of LDHA Y10 through FGFR1, elevating aerobic glycolysis of liver cancer cells (Wang et al., 2020). The tyrosine phosphorylation of LDHA is important for NADH/NAD^+^ redox homeostasis in cancer cells, due to a compensatory increase in mitochondrial respiration in Y10F cells (Fan et al., 2011).

It deserves to be mentioned that phosphorylation of LDHB has recently been found in p53-deficient cancer cells (Cheng et al., 2019). Aurora-A directly binds and phosphorylates LDHB, resulting in significantly rapid NAD + regeneration and upregulation of glycolysis. Together with LDHA, an interesting observation is that the phosphorylation of different types of LDH so far seems to promote glycolysis for NAD regeneration.

#### Acetylation accelerates degradation of LDHA but inhibits LDHB activity

LDHA is found acetylated at lysine 5 (K5) in pancreatic cancer cells, which reduces LDHA catalytic activity and decreases its protein level (Zhao et al., 2013a). The K5-acetylated LDH-A is recognized by the HSC70 chaperone and delivered to lysosomes for degradation. Replacement of endogenous LDHA with an acetylation mimetic mutant leads to a decrease in cancer cell proliferation and migration, indicating the critical role of LDHA acetylation in cell growth control (Zhao et al., 2013b). In this model, acetylation decreases the LDHA protein level and further affects the TCA cycle and oxidative phosphorylation for efficient energy production in cancer cells.

Even though four putative acetylation sites were identified in LDHB by mass spectrometry, only K329 has been identified as functional in cancers (Shi et al., 2019). SIRT5, which is demonstrated to directly deacetylate LDHB at lysine 329, increases LDHB activity and promotes autophagy in human colorectal cancer, leading to tumor growth and poor prognosis.

#### Succinylation maintains the stability of LDHA

Succinylation has recently been found to be another modification affecting LDHA stability. Proteomics analysis suggests LDHA is upregulated in gastric cancer tissues and highly succinylated on K222 (Li X. et al., 2020). CPT1A binds to and succinylates LDHA on K222, which inhibits ubiquitinated LDHA binding to SQSTM1. This modified pattern of lysosomal pathway inhibition increases the levels of LDHA, leading to poor prognosis in patients with GC.

#### Ubiquitination plays diverse roles in LDHA function

Ubiquitination is an essential posttranslational modification that regulates protein stability or function. Even though there are numerous predicted ubiquitination sites in human LDHA protein, as shown on the Phosphosite website (https://www.phosphosite.org) (Akimov et al., 2018), such as K14 and K118, few studies focus on that. A former study identifies mono-ubiquitinated LDHA in skeletal muscle cells exposed to oxidative stress (Onishi et al., 2005). In this study, H_2_O_2_ treatment specifically enhances the levels of mono-ubiquitinated LDHA and lysosomal catabolism of LDH, illustrating the regulated relationship between ROS and LDHA stability. The authors infer that LDHA may be mono-ubiquitinated without further extension of the poly-ubiquitination chain because mono-ubiquitination of LDHA may also mediate its sorting into the lysosome to enhance the degradation. Li X. et al. (2020), however, showed that poly-ubiquitination of LDHA exists and LDHA was mainly ubiquitinated by K63-linked ubiquitin, though the degradation of LDHA is not mediated by the proteasome pathway. Another recent research shows that CTLH E3 ligase mediates poly-ubiquitination of LDHA and PKM2 (Maitland et al., 2021). However, this ubiquitination inhibits the activity levels of enzymes but not the stability. Whether poly-ubiquitinated LDHA exists *in vivo* and its function needs further detection.

#### Methylation is associated with LDHA activity

A recent investigation shows that methylation of LDHA influences protein activity but not stability. Protein arginine methyltransferase 3 enhances arginine methylation of LDHA in HCC (Lei et al., 2022). LDHA could be methylated at R112, which is located at the nucleotide-binding Rossmann fold and near the catalytic loop (Woodford et al., 2020). Methylation at this residue might influence LDH activity because the growth of PRMT3-overexpressed cancer cells is affected by this site mutation. However, how methylation promotes LDHA function and whether this modification exists in other cancers remain a mystery.

## LDH in clinical translation

**FIGURE 3:**
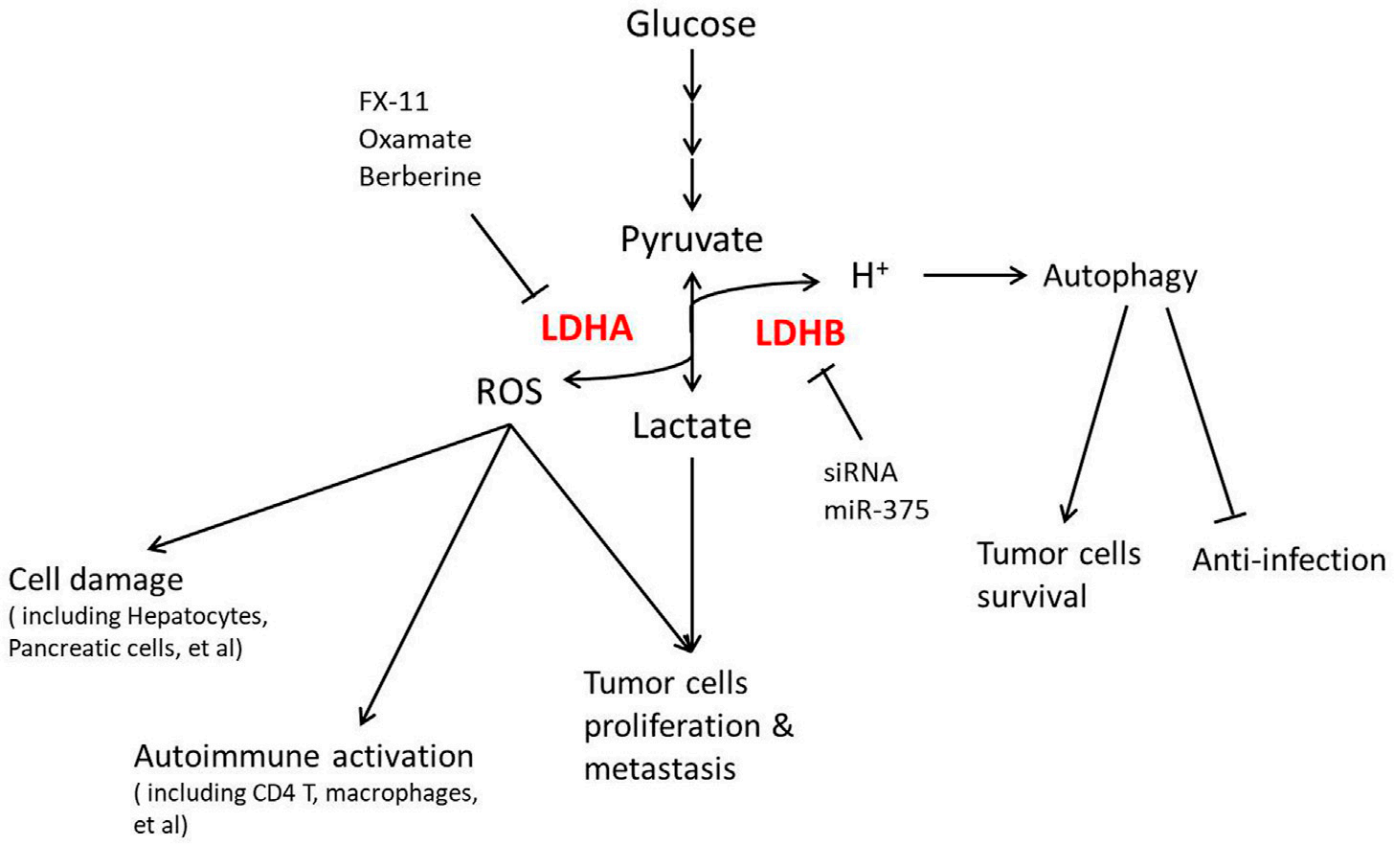
LDH regulation in diseases and clinical translation.

As discussed previously that LDH is involved in energy and redox regulation, more and more recent studies focus on LDH function in diseases, as shown in Figure 3. The Warburg effect was mainly reported in cancer, resulting in significant attention on LDH in cancer therapy, especially how different LDHA inhibiting methods show a positive effect. LDHA is overexpressed and associated with poor prognosis in non-small cell lung cancer (NSCLC), where LDHA inhibition by oxamate remarkably increased radiosensitivity through ROS accumulation and cellular ATP depletion (Yang et al., 2021). Targeting LDHA through miR-30a-5p could be a potential therapeutic strategy in breast cancer for its obvious effect in suppressing breast cancer metastasis (Li L. et al., 2017). As an LDHA inhibitor, berberine plays a critical role in suppressing LDHA/AMPK-mTOR-mediated pancreatic adenocarcinoma progression (Cheng et al., 2021). Galloflavin, which inhibits LDH, improves cell viability and survival in acute liver failure (Ferriero et al., 2018), suggesting that the LDHA inhibitor plays roles in not only cancer therapy. In consideration of the clinical permission of the last two inhibitors, LDHA inhibition might be a promising treatment in clinical practice.

Not only in cancer but also in metabolic syndromes including obesity and diabetes, LDHA is now found operating. LDHA is found upregulated in human islets during type 2 diabetes, which was mainly localized in human α-cells, while it is expressed at a very low level in β-cells, indicating a possible role of LDHA in the islet secretory dysfunction and diabetes progression (Sanchez et al., 2021). The expression and phosphorylation of LDHA might be involved in type 1 diabetes by β-cell apoptosis (Huang et al., 2021). Lactate production is highly increased during obesity (Lin et al., 2022), while hypothalamic LDHA is regulated by leptin signaling in obese rats (Abraham et al., 2018). Due to these effects, some researchers tried mediating LDH to intervene in metabolic syndromes. LDHA inhibitor oxamate significantly abolished the TGF-β1-induced expression of renal fibrosis-related proteins, which might be applied in the treatment of diabetic kidney disease (Lee et al., 2022). LDHA-mediated ROS generation in chondrocytes is now thought to be a pathogenic factor of osteoarthritis, where inhibiting LDHA with FX-11 is efficacious in articular chondrocytes (Arra et al., 2020). Unfortunately, there is still a lack of clinical applications of LDH inhibitors for metabolic diseases, which remains to be explored.

LDHB in cancer is also regarded as a target recently. In uterine cancer patients, high expression of MCT1, together with LDHB, predicts poor survival because LDHB actively controls lysosomal activity in oxidative cancer cells (Brisson et al., 2016). In this perspective, silencing LDHB decreases the cell number in most cancer cell lines *in vitro* and *in vivo*. R-2-hydroxyglutarate mediated anti-leukemia activity partly through LDHB inhibition in primary patient samples (Qing et al., 2021), supporting the inhibition of LDHB as a potential treatment for most cancer. However, the tissue selectivity of LDHB inhibition is key to clinical application due to its possible side effects on immune systems. LDHB inhibition facilitates the progression of classical swine fever virus (CSFV) infection resulting from its induction of cellular mitophagy (Fan et al., 2021). miR-375-mediated downregulation of macrophage LDHB skewed TAMs to function as a lactate and sterol source for proliferation of tumor cells (Frank et al., 2021), emphasizing that the targeting cells should be strictly in LDHB inhibition treatment.

In another aspect, the side effects of direct handling of lactate metabolism should be known. LDHA deficiency was first reported in the 1980s (Kanno et al., 1980), and the patients have been found to present with exertional myopathy, erythematosquamous skin lesions, and uterine stiffness during pregnancy (Lai et al., 2018). Though most symptoms were not shown under non-exercise conditions, LDHA deficiency in muscles still poses an enormous risk (Ariceta et al., 2021). LDHA deficiency cripples the cellular redox control and diminishes ATP production in effector T cells, resulting in attenuated PI3K signaling and defective antimicrobial immunity (Xu et al., 2021). LDHB inhibition causes lactate accumulation in some of the aforementioned research studies, which might result in cell apoptosis or necrosis in other organs (Lin et al., 2022). All these suggest the risk of application of LDH intervention, mainly the dose and the targeted cells for inhibition.

## Conclusions and perspective

Considering the relationship between metabolism and redox homeostasis, lactate homeostasis, associated with NAD/NADH regeneration, regulates cellular redox robustness. The lactate shuttle theory also suggests that lactate in circulation is a pool participating in systemic regulation. LDH is the direct regulator of lactate–pyruvate balance, the function of which is linked tightly to cellular oxidative stress. The transcriptional and post-translational regulation of LDHA adjusts the lactate–pyruvate ratio; through this way, the mutual effect between LDH and redox robustness is built.

Though the relationship between LDH and redox has been discussed for decades, there are still many questions that remain to be solved. Do the very differently expressed patterns of LDHA and LDHB suggest diverse regulatory modes among tissues? Most studies focus on LDHA, but not on LDHB, and how LDHB is regulated and its role in ROS regulation deserves discovery. Moreover, we still know little about the posttranslational regulation of LDH. Substantial modification of LDHA has been predicted or detected by MS, but the sites and functions that have been reported are limited. These questions, potentially, would promote our understanding of metabolic regulation on redox homeostasis.

## References

[B1] Abraham M. A. Rasti M. Bauer P. V. Lam T. K. T. (2018). Leptin enhances hypothalamic lactate dehydrogenase A (LDHA)-dependent glucose sensing to lower glucose production in high-fat-fed rats. J. Biol. Chem. 293, 4159–4166. 10.1074/jbc.RA117.000838 29374061 PMC5857974

[B2] Akimov V. Barrio-Hernandez I. Hansen S. V. F. Hallenborg P. Pedersen A. K. Bekker-Jensen D. B. (2018). UbiSite approach for comprehensive mapping of lysine and N-terminal ubiquitination sites. Nat. Struct. Mol. Biol. 25, 631–640. 10.1038/s41594-018-0084-y 29967540

[B3] Ariceta G. Barrios K. Brown B. D. Hoppe B. Rosskamp R. Langman C. B. (2021). Hepatic lactate dehydrogenase A: An RNA interference target for the treatment of all known types of primary hyperoxaluria. Kidney Int. Rep. 6, 1088–1098. 10.1016/j.ekir.2021.01.029 33912759 PMC8071644

[B4] Arra M. Swarnkar G. Ke K. Otero J. E. Ying J. Duan X. (2020). LDHA-mediated ROS generation in chondrocytes is a potential therapeutic target for osteoarthritis. Nat. Commun. 11, 3427. 10.1038/s41467-020-17242-0 32647171 PMC7347613

[B5] Augoff K. Hryniewicz-Jankowska A. Tabola R. (2015). Lactate dehydrogenase 5: An old friend and a new hope in the war on cancer. Cancer Lett. 358, 1–7. 10.1016/j.canlet.2014.12.035 25528630

[B6] Bellamacina C. R. (1996). The nicotinamide dinucleotide binding motif: A comparison of nucleotide binding proteins. FASEB J. 10, 1257–1269. 10.1096/fasebj.10.11.8836039 8836039

[B7] Blum A. Mostow K. Jackett K. Kelty E. Dakpa T. Ryan C. (2021). KLF4 regulates metabolic homeostasis in response to stress. Cells 10. 10.3390/cells10040830 PMC8067718 33917010

[B8] Brand K. A. Hermfisse U. (1997). Aerobic glycolysis by proliferating cells: A protective strategy against reactive oxygen species. FASEB J. 11, 388–395. 10.1096/fasebj.11.5.9141507 9141507

[B9] Brisson L. Banski P. Sboarina M. Dethier C. Danhier P. Fontenille M. J. (2016). Lactate dehydrogenase B controls lysosome activity and autophagy in cancer. Cancer Cell. 30, 418–431. 10.1016/j.ccell.2016.08.005 27622334

[B10] Brooks G. A. (2020). Lactate as a fulcrum of metabolism. Redox Biol. 35, 101454. 10.1016/j.redox.2020.101454 32113910 PMC7284908

[B11] Brooks G. A. (2018). The science and translation of lactate shuttle theory. Cell. Metab. 27, 757–785. 10.1016/j.cmet.2018.03.008 29617642

[B12] Brooks G. A. Dubouchaud H. Brown M. Sicurello J. P. Butz C. E. (1999). Role of mitochondrial lactate dehydrogenase and lactate oxidation in the intracellular lactate shuttle. Proc. Natl. Acad. Sci. U. S. A. 96, 1129–1134. 10.1073/pnas.96.3.1129 9927705 PMC15362

[B13] Cameron A. Read J. Tranter R. Winter V. J. Sessions R. B. Brady R. L. (2004). Identification and activity of a series of azole-based compounds with lactate dehydrogenase-directed anti-malarial activity. J. Biol. Chem. 279, 31429–31439. 10.1074/jbc.M402433200 15117937

[B14] Carriere A. Jeanson Y. Berger-Muller S. Andre M. Chenouard V. Arnaud E. (2014). Browning of white adipose cells by intermediate metabolites: An adaptive mechanism to alleviate redox pressure. Diabetes 63, 3253–3265. 10.2337/db13-1885 24789919

[B15] Cheng A. Zhang P. Wang B. Yang D. Duan X. Jiang Y. (2019). Aurora-A mediated phosphorylation of LDHB promotes glycolysis and tumor progression by relieving the substrate-inhibition effect. Nat. Commun. 10, 5566. 10.1038/s41467-019-13485-8 31804482 PMC6895051

[B16] Cheng C. S. Tan H. Y. Wang N. Chen L. Meng Z. Chen Z. (2021). Functional inhibition of lactate dehydrogenase suppresses pancreatic adenocarcinoma progression. Clin. Transl. Med. 11, e467. 10.1002/ctm2.467 34185423 PMC8238920

[B17] Cooper J. A. Esch F. S. Taylor S. S. Hunter T. (1984). Phosphorylation sites in enolase and lactate dehydrogenase utilized by tyrosine protein kinases *in vivo* and *in vitro* . J. Biol. Chem. 259, 7835–7841. 10.1016/s0021-9258(17)42869-9 6330085

[B18] Corbet C. Bastien E. Draoui N. Doix B. Mignion L. Jordan B. F. (2018). Interruption of lactate uptake by inhibiting mitochondrial pyruvate transport unravels direct antitumor and radiosensitizing effects. Nat. Commun. 9, 1208. 10.1038/s41467-018-03525-0 29572438 PMC5865202

[B19] Cui J. Shi M. Xie D. Wei D. Jia Z. Zheng S. (2014). FOXM1 promotes the warburg effect and pancreatic cancer progression via transactivation of LDHA expression. Clin. Cancer Res. 20, 2595–2606. 10.1158/1078-0432.CCR-13-2407 24634381 PMC4024335

[B20] Dang C. V. (2012). MYC on the path to cancer. Cell. 149, 22–35. 10.1016/j.cell.2012.03.003 22464321 PMC3345192

[B21] Daw C. C. Ramachandran K. Enslow B. T. Maity S. Bursic B. Novello M. J. (2020). Lactate elicits ER-mitochondrial Mg(2+) dynamics to integrate cellular metabolism. Cell. 183, 474–489. 10.1016/j.cell.2020.08.049 33035451 PMC7572828

[B22] Dong S. Liang S. Cheng Z. Zhang X. Luo L. Li L. (2022). ROS/PI3K/Akt and Wnt/β-catenin signalings activate HIF-1α-induced metabolic reprogramming to impart 5-fluorouracil resistance in colorectal cancer. J. Exp. Clin. Cancer Res. 41, 15. 10.1186/s13046-021-02229-6 34998404 PMC8742403

[B23] Fan J. Hitosugi T. Chung T. W. Xie J. Ge Q. Gu T. L. (2011). Tyrosine phosphorylation of lactate dehydrogenase A is important for NADH/NAD(+) redox homeostasis in cancer cells. Mol. Cell. Biol. 31, 4938–4950. 10.1128/MCB.06120-11 21969607 PMC3233034

[B24] Fan S. Wu K. Zhao M. Yuan J. Ma S. Zhu E. (2021). LDHB inhibition induces mitophagy and facilitates the progression of CSFV infection. Autophagy 17, 2305–2324. 10.1080/15548627.2020.1823123 32924761 PMC8496725

[B25] Fantin V. R. St-Pierre J. Leder P. (2006). Attenuation of LDH-A expression uncovers a link between glycolysis, mitochondrial physiology, and tumor maintenance. Cancer Cell. 9, 425–434. 10.1016/j.ccr.2006.04.023 16766262

[B26] Feng Y. Xiong Y. Qiao T. Li X. Jia L. Han Y. (2018). Lactate dehydrogenase A: A key player in carcinogenesis and potential target in cancer therapy. Cancer Med. 7, 6124–6136. 10.1002/cam4.1820 30403008 PMC6308051

[B27] Ferriero R. Nusco E. De Cegli R. Carissimo A. Manco G. Brunetti-Pierri N. (2018). Pyruvate dehydrogenase complex and lactate dehydrogenase are targets for therapy of acute liver failure. J. Hepatol. 69, 325–335. 10.1016/j.jhep.2018.03.016 29580866 PMC6057136

[B28] Frank A. C. Raue R. Fuhrmann D. C. Sirait-Fischer E. Reuse C. Weigert A. (2021). Lactate dehydrogenase B regulates macrophage metabolism in the tumor microenvironment. Theranostics 11, 7570–7588. 10.7150/thno.58380 34158867 PMC8210612

[B29] Ganapathy-Kanniappan S. Geschwind J. F. (2013). Tumor glycolysis as a target for cancer therapy: Progress and prospects. Mol. Cancer 12, 152. 10.1186/1476-4598-12-152 24298908 PMC4223729

[B30] Goldberg E. (1971). Immunochemical specificity of lactate dehydrogenase-X. Proc. Natl. Acad. Sci. U. S. A. 68, 349–352. 10.1073/pnas.68.2.349 5277084 PMC388935

[B31] Gordan J. D. Thompson C. B. Simon M. C. (2007). HIF and c-myc: Sibling rivals for control of cancer cell metabolism and proliferation. Cancer Cell. 12, 108–113. 10.1016/j.ccr.2007.07.006 17692803 PMC3215289

[B32] Granchi C. Bertini S. Macchia M. Minutolo F. (2010). Inhibitors of lactate dehydrogenase isoforms and their therapeutic potentials. Curr. Med. Chem. 17, 672–697. 10.2174/092986710790416263 20088761

[B33] Granchi C. Calvaresi E. C. Tuccinardi T. Paterni I. Macchia M. Martinelli A. (2013). Assessing the differential action on cancer cells of LDH-A inhibitors based on the N-hydroxyindole-2-carboxylate (NHI) and malonic (Mal) scaffolds. Org. Biomol. Chem. 11, 6588–6596. 10.1039/c3ob40870a 23986182 PMC3828658

[B34] Grist J. T. Jarvis L. B. Georgieva Z. Thompson S. Kaur Sandhu H. Burling K. (2018). Extracellular lactate: A novel measure of T cell proliferation. J. Immunol. 200, 1220–1226. 10.4049/jimmunol.1700886 29288205 PMC5776880

[B35] Grosse F. Nasheuer H. P. Scholtissek S. Schomburg U. (1986). Lactate dehydrogenase and glyceraldehyde-phosphate dehydrogenase are single-stranded DNA-binding proteins that affect the DNA-polymerase-alpha-primase complex. Eur. J. Biochem. 160, 459–467. 10.1111/j.1432-1033.1986.tb10062.x 3536507

[B36] Halestrap A. P. (2013). The SLC16 gene family - structure, role and regulation in health and disease. Mol. Asp. Med. 34, 337–349. 10.1016/j.mam.2012.05.003 23506875

[B37] Halestrap A. P. Wilson M. C. (2012). The monocarboxylate transporter family-role and regulation. IUBMB Life 64, 109–119. 10.1002/iub.572 22162139

[B38] Handy D. E. Loscalzo J. (2012). Redox regulation of mitochondrial function. Antioxid. Redox Signal. 16, 1323–1367. 10.1089/ars.2011.4123 22146081 PMC3324814

[B39] Hashimoto T. Hussien R. Brooks G. A. (2006). Colocalization of MCT1, CD147, and LDH in mitochondrial inner membrane of L6 muscle cells: Evidence of a mitochondrial lactate oxidation complex. Am. J. Physiol. Endocrinol. Metab. 290, E1237–E1244. 10.1152/ajpendo.00594.2005 16434551

[B40] Hashimoto T. Hussien R. Oommen S. Gohil K. Brooks G. A. (2007). Lactate sensitive transcription factor network in L6 cells: Activation of MCT1 and mitochondrial biogenesis. FASEB J. 21, 2602–2612. 10.1096/fj.07-8174com 17395833

[B41] Haugrud A. B. Zhuang Y. Coppock J. D. Miskimins W. K. (2014). Dichloroacetate enhances apoptotic cell death via oxidative damage and attenuates lactate production in metformin-treated breast cancer cells. Breast Cancer Res. Treat. 147, 539–550. 10.1007/s10549-014-3128-y 25212175 PMC4184194

[B42] Hirschhaeuser F. Sattler U. G. Mueller-Klieser W. (2011). Lactate: A metabolic key player in cancer. Cancer Res. 71, 6921–6925. 10.1158/0008-5472.CAN-11-1457 22084445

[B43] Huang Y. N. Chiang S. L. Lin Y. J. Liu S. C. Li Y. H. Liao Y. C. (2021). Long, noncoding RNA SRA induces apoptosis of beta-cells by promoting the IRAK1/LDHA/lactate pathway. Int. J. Mol. Sci. 22, 1720. 10.3390/ijms22041720 33572095 PMC7914996

[B44] Hui S. Ghergurovich J. M. Morscher R. J. Jang C. Teng X. Lu W. (2017). Glucose feeds the TCA cycle via circulating lactate. Nature 551, 115–118. 10.1038/nature24057 29045397 PMC5898814

[B45] Huynh D. T. N. Jin Y. Van Nguyen D. Myung C. S. Heo K. S. (2022). Ginsenoside Rh1 inhibits angiotensin II-induced vascular smooth muscle cell migration and proliferation through suppression of the ROS-mediated ERK1/2/p90RSK/KLF4 signaling pathway. Antioxidants (Basel) 11, 643. 10.3390/antiox11040643 35453328 PMC9030830

[B46] Iyanagi T. (2019). Molecular mechanism of metabolic NAD(P)H-dependent electron-transfer systems: The role of redox cofactors. Biochim. Biophys. Acta. Bioenerg. 1860, 233–258. 10.1016/j.bbabio.2018.11.014 30419202

[B47] Jeong D. W. Cho I. T. Kim T. S. Bae G. W. Kim I. H. Kim I. Y. (2006). Effects of lactate dehydrogenase suppression and glycerol-3-phosphate dehydrogenase overexpression on cellular metabolism. Mol. Cell. Biochem. 284, 1–8. 10.1007/s11010-005-9004-7 16477389

[B48] Ji Y. Yang C. Tang Z. Yang Y. Tian Y. Yao H. (2017). Corrigendum: Adenylate kinase hCINAP determines self-renewal of colorectal cancer stem cells by facilitating LDHA phosphorylation. Nat. Commun. 8, 16000. 10.1038/ncomms16000 28621309 PMC5481747

[B49] Jiang W. Zhou F. Li N. Li Q. Wang L. (2015). FOXM1-LDHA signaling promoted gastric cancer glycolytic phenotype and progression. Int. J. Clin. Exp. Pathol. 8, 6756–6763. 26261559 PMC4525893

[B50] Kachel P. Trojanowicz B. Sekulla C. Prenzel H. Dralle H. Hoang-Vu C. (2015). Phosphorylation of pyruvate kinase M2 and lactate dehydrogenase A by fibroblast growth factor receptor 1 in benign and malignant thyroid tissue. BMC Cancer 15, 140. 10.1186/s12885-015-1135-y 25880801 PMC4393606

[B51] Kanno T. Sudo K. Takeuchi I. Kanda S. Honda N. Nishimura Y. (1980). Hereditary deficiency of lactate dehydrogenase M-subunit. Clin. Chim. Acta. 108, 267–276. 10.1016/0009-8981(80)90013-3 7449146

[B52] Kopanja D. Pandey A. Kiefer M. Wang Z. Chandan N. Carr J. R. (2015). Essential roles of FoxM1 in Ras-induced liver cancer progression and in cancer cells with stem cell features. J. Hepatol. 63, 429–436. 10.1016/j.jhep.2015.03.023 25828473 PMC4508215

[B53] Krieg A. F. Rosenblum L. J. Henry J. B. (1967). Lactate dehydrogenase isoenzymes. Clin. Chem. 13, 196–203. 10.1093/clinchem/13.3.196 6018717

[B54] Lagarde D. Jeanson Y. Portais J. C. Galinier A. Ader I. Casteilla L. (2021). Lactate fluxes and plasticity of adipose tissues: A redox perspective. Front. Physiol. 12, 689747. 10.3389/fphys.2021.689747 34276410 PMC8278056

[B55] Lai C. Pursell N. Gierut J. Saxena U. Zhou W. Dills M. (2018). Specific inhibition of hepatic lactate dehydrogenase reduces oxalate production in mouse models of primary hyperoxaluria. Mol. Ther. 26, 1983–1995. 10.1016/j.ymthe.2018.05.016 29914758 PMC6094358

[B56] Le A. Cooper C. R. Gouw A. M. Dinavahi R. Maitra A. Deck L. M. (2010). Inhibition of lactate dehydrogenase A induces oxidative stress and inhibits tumor progression. Proc. Natl. Acad. Sci. U. S. A. 107, 2037–2042. 10.1073/pnas.0914433107 20133848 PMC2836706

[B57] Lee D. Y. Kim J. Y. Ahn E. Hyeon J. S. Kim G. H. Park K. J. (2022). Associations between local acidosis induced by renal LDHA and renal fibrosis and mitochondrial abnormalities in patients with diabetic kidney disease. Transl. Res. 249, 88–109. 10.1016/j.trsl.2022.06.015 35788054

[B58] Lei Y. Han P. Chen Y. Wang H. Wang S. Wang M. (2022). Protein arginine methyltransferase 3 promotes glycolysis and hepatocellular carcinoma growth by enhancing arginine methylation of lactate dehydrogenase A. Clin. Transl. Med. 12, e686. 10.1002/ctm2.686 35090076 PMC8797063

[B59] Li G. Xie C. Lu S. Nichols R. G. Tian Y. Li L. (2017a). Intermittent fasting promotes white adipose browning and decreases obesity by shaping the gut microbiota. Cell. Metab. 26, 672–685. 10.1016/j.cmet.2017.08.019 28918936 PMC5668683

[B60] Li L. Kang L. Zhao W. Feng Y. Liu W. Wang T. (2017b). miR-30a-5p suppresses breast tumor growth and metastasis through inhibition of LDHA-mediated Warburg effect. Cancer Lett. 400, 89–98. 10.1016/j.canlet.2017.04.034 28461244

[B61] Li S. Ding J. Jiang L. Hayat M. A. Song Q. Li Y. (2020a). Dynamic ROS production and gene expression of heifers blood neutrophil in a oligofructose overload model. Front. Vet. Sci. 7, 211. 10.3389/fvets.2020.00211 32373641 PMC7186304

[B62] Li X. Zhang C. Zhao T. Su Z. Li M. Hu J. (2020b). Lysine-222 succinylation reduces lysosomal degradation of lactate dehydrogenase a and is increased in gastric cancer. J. Exp. Clin. Cancer Res. 39, 172. 10.1186/s13046-020-01681-0 32859246 PMC7455916

[B63] Liberti M. V. Locasale J. W. (2016). The warburg effect: How does it benefit cancer cells? Trends biochem. Sci. 41, 211–218. 10.1016/j.tibs.2015.12.001 26778478 PMC4783224

[B64] Lim S. Liu H. Madeira da Silva L. Arora R. Liu Z. Phillips J. B. (2016). Immunoregulatory protein B7-H3 reprograms glucose metabolism in cancer cells by ROS-mediated stabilization of HIF1α. Cancer Res. 76, 2231–2242. 10.1158/0008-5472.CAN-15-1538 27197253 PMC4874665

[B65] Lin Y. Bai M. Wang S. Chen L. Li Z. Li C. (2022). Lactate is a key mediator that links obesity to insulin resistance via modulating cytokine production from adipose tissue. Diabetes 71, 637–652. 10.2337/db21-0535 35044451

[B66] Liu Y. Guo J. Z. Liu Y. Wang K. Ding W. Wang H. (2018). Nuclear lactate dehydrogenase A senses ROS to produce alpha-hydroxybutyrate for HPV-induced cervical tumor growth. Nat. Commun. 9, 4429. 10.1038/s41467-018-06841-7 30356100 PMC6200739

[B67] Locasale J. W. Cantley L. C. (2011). Metabolic flux and the regulation of mammalian cell growth. Cell. Metab. 14, 443–451. 10.1016/j.cmet.2011.07.014 21982705 PMC3196640

[B68] Lu J. Tan M. Cai Q. (2015). The warburg effect in tumor progression: Mitochondrial oxidative metabolism as an anti-metastasis mechanism. Cancer Lett. 356, 156–164. 10.1016/j.canlet.2014.04.001 24732809 PMC4195816

[B69] Maitland M. E. R. Kuljanin M. Wang X. Lajoie G. A. Schild-Poulter C. (2021). Proteomic analysis of ubiquitination substrates reveals a CTLH E3 ligase complex-dependent regulation of glycolysis. FASEB J. 35, e21825. 10.1096/fj.202100664R 34383978 PMC9292413

[B70] Majmundar A. J. Wong W. J. Simon M. C. (2010). Hypoxia-inducible factors and the response to hypoxic stress. Mol. Cell. 40, 294–309. 10.1016/j.molcel.2010.09.022 20965423 PMC3143508

[B71] Mintun M. A. Vlassenko A. G. Rundle M. M. Raichle M. E. (2004). Increased lactate/pyruvate ratio augments blood flow in physiologically activated human brain. Proc. Natl. Acad. Sci. U. S. A. 101, 659–664. 10.1073/pnas.0307457100 14704276 PMC327204

[B72] Neilands J. B. (1952). The purity of crystalline lactic dehydrogenase. Science 115, 143–144. 10.1126/science.115.2980.143 14913194

[B73] Onishi Y. Hirasaka K. Ishihara I. Oarada M. Goto J. Ogawa T. (2005). Identification of mono-ubiquitinated LDH-A in skeletal muscle cells exposed to oxidative stress. Biochem. Biophys. Res. Commun. 336, 799–806. 10.1016/j.bbrc.2005.08.175 16154111

[B74] Park H. J. Carr J. R. Wang Z. Nogueira V. Hay N. Tyner A. L. (2009). FoxM1, a critical regulator of oxidative stress during oncogenesis. EMBO J. 28, 2908–2918. 10.1038/emboj.2009.239 19696738 PMC2760115

[B75] Passarella S. Paventi G. Pizzuto R. (2014). The mitochondrial L-lactate dehydrogenase affair. Front. Neurosci. 8, 407. 10.3389/fnins.2014.00407 25538557 PMC4260494

[B76] Qing Y. Dong L. Gao L. Li C. Li Y. Han L. (2021). R-2-hydroxyglutarate attenuates aerobic glycolysis in leukemia by targeting the FTO/m(6)A/PFKP/LDHB axis. Mol. Cell. 81, 922–939.e9. 10.1016/j.molcel.2020.12.026 33434505 PMC7935770

[B77] Quinn W. J. 3rd Jiao J. TeSlaa T. Stadanlick J. Wang Z. Wang L. (2020). Lactate limits T cell proliferation via the NAD(H) redox state. Cell. Rep. 33, 108500. 10.1016/j.celrep.2020.108500 33326785 PMC7830708

[B78] Rabinowitz J. D. Enerback S. (2020). Lactate: The ugly duckling of energy metabolism. Nat. Metab. 2, 566–571. 10.1038/s42255-020-0243-4 32694798 PMC7983055

[B79] Rashmi R. Huang X. Floberg J. M. Elhammali A. E. McCormick M. L. Patti G. J. (2018). Radioresistant cervical cancers are sensitive to inhibition of glycolysis and redox metabolism. Cancer Res. 78, 1392–1403. 10.1158/0008-5472.CAN-17-2367 29339540 PMC5856626

[B80] Sanchez P. K. M. Khazaei M. Gatineau E. Geravandi S. Lupse B. Liu H. (2021). LDHA is enriched in human islet alpha cells and upregulated in type 2 diabetes. Biochem. Biophys. Res. Commun. 568, 158–166. 10.1016/j.bbrc.2021.06.065 34217973 PMC8364499

[B81] Sattler U. G. Mueller-Klieser W. (2009). The anti-oxidant capacity of tumour glycolysis. Int. J. Radiat. Biol. 85, 963–971. 10.3109/09553000903258889 19895273

[B82] Sheng S. L. Liu J. J. Dai Y. H. Sun X. G. Xiong X. P. Huang G. (2012). Knockdown of lactate dehydrogenase A suppresses tumor growth and metastasis of human hepatocellular carcinoma. FEBS J. 279, 3898–3910. 10.1111/j.1742-4658.2012.08748.x 22897481

[B83] Shi L. Yan H. An S. Shen M. Jia W. Zhang R. (2019). SIRT5-mediated deacetylation of LDHB promotes autophagy and tumorigenesis in colorectal cancer. Mol. Oncol. 13, 358–375. 10.1002/1878-0261.12408 30443978 PMC6360364

[B84] Shi M. Cui J. Du J. Wei D. Jia Z. Zhang J. (2014). A novel KLF4/LDHA signaling pathway regulates aerobic glycolysis in and progression of pancreatic cancer. Clin. Cancer Res. 20, 4370–4380. 10.1158/1078-0432.CCR-14-0186 24947925 PMC4134726

[B85] Shim H. Dolde C. Lewis B. C. Wu C. S. Dang G. Jungmann R. A. (1997). c-Myc transactivation of LDH-A: implications for tumor metabolism and growth. Proc. Natl. Acad. Sci. U. S. A. 94, 6658–6663. 10.1073/pnas.94.13.6658 9192621 PMC21214

[B86] Su X. Yang Y. Yang Q. Pang B. Sun S. Wang Y. (2021). NOX4-derived ROS-induced overexpression of FOXM1 regulates aerobic glycolysis in glioblastoma. BMC Cancer 21, 1181. 10.1186/s12885-021-08933-y 34740322 PMC8571893

[B87] Talaiezadeh A. Shahriari A. Tabandeh M. R. Fathizadeh P. Mansouri S. (2015). Kinetic characterization of lactate dehydrogenase in normal and malignant human breast tissues. Cancer Cell. Int. 15, 19. 10.1186/s12935-015-0171-7 25705126 PMC4334850

[B88] Tauffenberger A. Fiumelli H. Almustafa S. Magistretti P. J. (2019). Lactate and pyruvate promote oxidative stress resistance through hormetic ROS signaling. Cell. Death Dis. 10, 653. 10.1038/s41419-019-1877-6 31506428 PMC6737085

[B89] Theret M. Gsaier L. Schaffer B. Juban G. Ben Larbi S. Weiss-Gayet M. (2017). AMPKα1-LDH pathway regulates muscle stem cell self-renewal by controlling metabolic homeostasis. EMBO J. 36, 1946–1962. 10.15252/embj.201695273 28515121 PMC5494470

[B90] Tian C. Kim Y. J. Hali S. Choo O. S. Lee J. S. Jung S. K. (2020). Suppressed expression of LDHB promotes age-related hearing loss via aerobic glycolysis. Cell. Death Dis. 11, 375. 10.1038/s41419-020-2577-y 32415082 PMC7229204

[B91] Walls J. F. Subleski J. J. Palmieri E. M. Gonzalez-Cotto M. Gardiner C. M. McVicar D. W. (2020). Metabolic but not transcriptional regulation by PKM2 is important for natural killer cell responses. Elife 9, e59166. 10.7554/eLife.59166 32812866 PMC7467725

[B92] Wang C. Li Y. Yan S. Wang H. Shao X. Xiao M. (2020). Interactome analysis reveals that lncRNA HULC promotes aerobic glycolysis through LDHA and PKM2. Nat. Commun. 11, 3162. 10.1038/s41467-020-16966-3 32572027 PMC7308313

[B93] Wang Y. Stancliffe E. Fowle-Grider R. Wang R. Wang C. Schwaiger-Haber M. (2022). Saturation of the mitochondrial NADH shuttles drives aerobic glycolysis in proliferating cells. Mol. Cell. 82, 3270–3283.e9. 10.1016/j.molcel.2022.07.007 35973426 PMC10134440

[B94] Watson M. J. Vignali P. D. A. Mullett S. J. Overacre-Delgoffe A. E. Peralta R. M. Grebinoski S. (2021). Metabolic support of tumour-infiltrating regulatory T cells by lactic acid. Nature 591, 645–651. 10.1038/s41586-020-03045-2 33589820 PMC7990682

[B95] Willson J. A. Arienti S. Sadiku P. Reyes L. Coelho P. Morrison T. (2022). Neutrophil HIF-1α stabilization is augmented by mitochondrial ROS produced via the glycerol 3-phosphate shuttle. Blood 139, 281–286. 10.1182/blood.2021011010 34411229 PMC8832465

[B96] Woodford M. R. Chen V. Z. Backe S. J. Bratslavsky G. Mollapour M. (2020). Structural and functional regulation of lactate dehydrogenase-A in cancer. Future Med. Chem. 12, 439–455. 10.4155/fmc-2019-0287 32064930

[B97] Wu G. Yang L. Xu Y. Jiang X. Jiang X. Huang L. (2018). FABP4 induces asthmatic airway epithelial barrier dysfunction via ROS-activated FoxM1. Biochem. Biophys. Res. Commun. 495, 1432–1439. 10.1016/j.bbrc.2017.11.106 29158087

[B98] Wu H. Wang Y. Ying M. Jin C. Li J. Hu X. (2021). Lactate dehydrogenases amplify reactive oxygen species in cancer cells in response to oxidative stimuli. Signal Transduct. Target. Ther. 6, 242. 10.1038/s41392-021-00595-3 34176927 PMC8236487

[B99] Xiao W. Wang R. S. Handy D. E. Loscalzo J. (2018). NAD(H) and NADP(H) redox couples and cellular energy metabolism. Antioxid. Redox Signal. 28, 251–272. 10.1089/ars.2017.7216 28648096 PMC5737637

[B100] Xie H. Hanai J. Ren J. G. Kats L. Burgess K. Bhargava P. (2014). Targeting lactate dehydrogenase-a inhibits tumorigenesis and tumor progression in mouse models of lung cancer and impacts tumor-initiating cells. Cell. Metab. 19, 795–809. 10.1016/j.cmet.2014.03.003 24726384 PMC4096909

[B101] Xu K. Yin N. Peng M. Stamatiades E. G. Shyu A. Li P. (2021). Glycolysis fuels phosphoinositide 3-kinase signaling to bolster T cell immunity. Science 371, 405–410. 10.1126/science.abb2683 33479154 PMC8380312

[B102] Yang Y. Chong Y. Chen M. Dai W. Zhou X. Ji Y. (2021). Targeting lactate dehydrogenase a improves radiotherapy efficacy in non-small cell lung cancer: From bedside to bench. J. Transl. Med. 19, 170. 10.1186/s12967-021-02825-2 33902615 PMC8074241

[B103] Zhang D. Tang Z. Huang H. Zhou G. Cui C. Weng Y. (2019). Metabolic regulation of gene expression by histone lactylation. Nature 574, 575–580. 10.1038/s41586-019-1678-1 31645732 PMC6818755

[B104] Zhao D. Xiong Y. Lei Q. Y. Guan K. L. (2013a). LDH-A acetylation: Implication in cancer. Oncotarget 4, 802–803. 10.18632/oncotarget.1007 23868819 PMC3757233

[B105] Zhao D. Zou S. W. Liu Y. Zhou X. Mo Y. Wang P. (2013b). Lysine-5 acetylation negatively regulates lactate dehydrogenase A and is decreased in pancreatic cancer. Cancer Cell. 23, 464–476. 10.1016/j.ccr.2013.02.005 23523103 PMC3885615

[B106] Zhao Y. Li M. Yao X. Fei Y. Lin Z. Li Z. (2020). HCAR1/MCT1 regulates tumor ferroptosis through the lactate-mediated AMPK-SCD1 activity and its therapeutic implications. Cell. Rep. 33, 108487. 10.1016/j.celrep.2020.108487 33296645

